# What COVID-19 Vaccination Strategy Should Be Implemented and Which Vaccines Should Be Used in the Post-Pandemic Era?

**DOI:** 10.3390/vaccines12101180

**Published:** 2024-10-17

**Authors:** Pedro Plans-Rubió

**Affiliations:** College of Physicians of Barcelona, 08017 Barcelona, Spain; pedro.plans@yahoo.es

**Keywords:** COVID-19 vaccination, adapted COVID-19 vaccines, universal vaccination, high-risk vaccination, herd immunity, seasonal vaccination, COVID-19 vaccination strategies, cost-effectiveness

## Abstract

COVID-19 vaccines have reduced the negative health and economic impact of the COVID-19 pandemic by preventing severe disease, hospitalizations and deaths. In the new socio-economic normality, the COVID-19 vaccination strategy can be universal or high-risk and seasonal or not seasonal, and different vaccines can be used. The universal vaccination strategy can achieve greater health and herd immunity effects and is associated with greater costs than the high-risk vaccination strategy. In each country, the optimal COVID-19 vaccination strategy must be decided by considering the advantages and disadvantages and assessing the costs, health effects and cost-effectiveness of the universal and high-risk vaccination strategies. The universal vaccination strategy should be implemented when the objective of the vaccination program is to achieve the greatest health benefits from COVID-19 vaccination and when its incremental cost-effectiveness ratio is lower than EUR 30,000−50,000 per QALY or LYG. The use of adapted vaccines targeting currently circulating variants of SARS-CoV-2 is necessary to avoid the immune escape of emerging variants.

## 1. Introduction

The COVID-19 vaccination programs developed since 2020 have been the cornerstone in preventing and controlling the pandemic associated with severe acute respiratory syndrome coronavirus-2 (SARS-CoV-2) [1]. Vaccination programs developed in all countries prevented millions of cases, hospitalizations and deaths, thus reducing its negative health, economic and social effects worldwide [2].

After the World Health Organization (WHO)’s declaration that COVID-19 was no longer a global health emergency on 5 May 2023 [3], the daily number of new confirmed COVID-19 cases has decreased worldwide [4]. Although no new COVID-19 waves have occurred since February 2023, cases, hospitalizations and deaths due to COVID-19 continue to occur in many countries. In the two-week period ending 18 August 2024, 119,663 confirmed cases were reported worldwide [4].

In the current epidemiological situation, it is therefore necessary to answer three questions: (1) Should COVID-19 vaccinations be maintained? (2) Should a high-risk or universal COVID-19 vaccination strategy be implemented? (3) Which COVID-19 vaccines should be used?

## 2. COVID-19 Vaccination

Concerning the first question, there are three reasons why COVID-19 vaccination programs should be implemented in all countries. First, COVID-19 can be an endemic disease with cases, hospitalizations and deaths occurring among vulnerable population groups. Second, vaccine-induced immunity wanes over time regardless of the vaccination regimen [5]. Third, emerging SARS-CoV-2 variants can reduce the effectiveness of vaccines in targeting prior variants [6]. WHO-Europe proposed the development of a transition plan with the objective of strengthening health emergency preparedness, response and resilience, and recommended the inclusion of the COVID-19 vaccination in other routine vaccination activities against respiratory viruses [7].

The answer to the second question depends on the epidemiological situation, available resources, available vaccines, health impact objectives and herd immunity objectives. In each country, the following options should be considered when deciding which vaccination strategy to implement: (1) continuous or seasonal vaccination; (2) universal or high-risk vaccination; and (3) the use of original, adapted or updated adapted vaccines. Decisions concerning these options are of great importance because they are associated with different costs, health impacts and herd immunity effects.

Most countries are moving toward seasonal COVID-19 vaccination strategies that consist of annual booster vaccinations. In the seasonal strategy, COVID-19 vaccinations are administered before the SARS-CoV-2 season, like influenza vaccination campaigns. This strategy is adequate from a logistics point of view, as the COVID-19 vaccine and other vaccines against respiratory viruses could be administered using the same organizational and human resources before the winter season. Nevertheless, such a seasonal vaccination strategy would only be adequate if SARS-CoV-2 infections followed a seasonal pattern [8]. The seasonal vaccination strategy requires intensive vaccination activities during the vaccination period (autumn/winter), and it can be difficult to achieve high percentages of COVID-19 vaccination coverage using this strategy. In 2009, the Council of the European Union recommended that all European Union Member States should achieve a 75% vaccination coverage with the seasonal influenza vaccine [9]. During the 2021–2022 season, all countries provided seasonal influenza vaccinations for adults aged ≥65 years and aged <65 years with risk factors, and three countries also provided universal influenza vaccinations for adults aged ≥18 years [9]. However, in Europe, the influenza vaccination coverage achieved in the 2020−2021 season was only 20% among all adults and 59% among adults aged 65 years or more [9].

In the United States, the Center for Disease Control and Prevention (CDC) has recommended the 2024−2025 COVID-19 vaccine for all individuals aged 6 months and older [10]. In Australia, the Department of Health and Age Care has recommended booster doses of COVID-19 vaccines for individuals aged 18–64 years every 12 months and to individuals aged 64 years and older every 6–12 months [11].

In the European Union and European Economic Area, all 30 countries recommend primary vaccination for all individuals aged ≥12 years [12]. However, universal and high-risk booster vaccination strategies were used for child, adolescent and adult immunization in the 2022–2023 vaccination campaign (Table 1). Universal booster vaccination strategies were used for children aged 5–11 years in four countries, for adolescents aged 12–17-years in seventeen countries, for adults aged ≥18 years in eight countries and for adults aged ≥30 years in one country [12] (Table 1). By contrast, high-risk booster vaccination strategies were used for children aged 5–11 years in 13 countries, for adolescents aged 12–17-years in 9 countries and for adults aged ≥18 years in 21 countries [12] (Table 1).

However, in March 2023, most countries of the European Union and European Economic Area were still discussing their future COVID-19 vaccination strategies for 2024 [12].

## 3. Comparison of the High-Risk and Universal COVID-19 Vaccination Strategies

The COVID-19 vaccination strategy implemented in each country can evolve depending on several factors, including the epidemic situation, the effectiveness of previously administered vaccinations, the efficacy and effectiveness of booster vaccines, the availability of adapted vaccines, the identification of risk groups, vaccination costs and vaccination coverage and herd immunity objectives.

The COVID-19 vaccination strategy can be either universal or high-risk, independently of whether COVID-19 vaccination is seasonal or not. The high-risk strategy focuses on immunizing individuals who have a greater risk of developing COVID-19 complications, while the universal vaccination strategy focuses on immunizing the whole population. The objective of the high-risk strategy is to prevent the negative health effects that can result from SARS-CoV-2 exposure among individuals belonging to high-risk population groups.

The high-risk COVID-19 vaccination strategy includes the following population groups: individuals aged ≥65 years (or other age indication); individuals who have heart disease, diabetes mellitus, chronic lung disease, obesity, chronic kidney disease, chronic liver disease, cancer, asthma, cerebrovascular disease or immunodeficiency; patients in long-term care facilities; and pregnant women [12,13]. Healthcare workers and personnel working in long-term care facilities are also included in the high-risk strategy [12,13].

The universal vaccination strategy was necessary during 2020–2022 because the whole population was susceptible to SARS-CoV-2. However, it can be difficult in the current epidemiological situation to decide between the universal and high-risk strategies because many individuals have received COVID-19 vaccines. As of 18 August 2024, the global percentages of COVID-19 vaccination coverage were 65.1% for full vaccination, 35.6% for booster doses and 70.7% for at least one vaccine dose [4]. Consequently, the decision between the universal and the high-risk vaccination strategies must be based on their advantages and disadvantages.

Compared with the universal COVID-19 vaccination strategy, the high-risk strategy has several advantages (Table 2). It includes only individuals who can obtain the greatest benefits from COVID-19 prevention. It can avoid the adverse effects of COVID-19 vaccination among individuals who are not deemed high-risk. COVID-19 vaccination can be included among other medical interventions for individuals with medical conditions with a high risk of COVID-19 complications.

However, the universal vaccination strategy has several advantages (Table 2). Vaccination activities can be developed without identifying who must be vaccinated and without implementing communication activities. Unlike the high-risk strategy, it can reduce the risk of exposure to SARS-CoV-2 infections in the population. The universal vaccination strategy can achieve greater global health effects than the high-risk strategy by (1) preventing COVID-19 complications among individuals without risk factors but who have a moderate or low risk of complications and (2) reducing SARS-CoV-2 exposure among all individuals.

The high-risk vaccination strategy is associated with lower costs than the universal vaccination strategy because it focuses on immunizing only individuals with risk factors (Table 2). In the United States, 75.4% of the population aged 18 years and over and 69.2% of the population aged 18−64 years have an increased risk of developing COVID-19 complications [14]. However, the universal vaccination strategy can achieve greater global health effects than the high-risk strategy (Table 2). A study comparing the universal and high-risk COVID-19 vaccination strategies implemented in individuals aged 10–19 years in Japan and South Korea found that the high-risk strategy was associated with 149% and 158% greater COVID-19 death rates in individuals aged 0–9 years and 10–19 years, respectively, than the universal vaccination [15]. The death rates among individuals aged 0–9 years and 10–19 years in South Korea were 2.49 (95% CI: 1.59–3.90) times and 2.58 (95% CI: 1.64–4.04) times greater, respectively, than in Japan [15].

The greater global health effects achieved by the universal COVID-19 vaccination strategy can be explained by two factors: (1) the prevention of COVID-19 complications among individuals without risk factors but with a moderate or low risk of complications, and (2) the herd immunity-associated prevention of SARS-CoV-2 infections and their complications among all individuals (Table 2). The universal vaccination strategy can prevent SARS-CoV-2 infections and their complications in all individuals because it can generate a prevalence of vaccine-induced protected individuals in the population that is greater than the prevalence required to establish herd immunity and block SARS-CoV-2 transmission [8,16]. However, vaccination effectiveness must be greater than 80% and the vaccination coverage must be greater than 70% to establish herd immunity against SARS-CoV-2 with greater transmissibility [8].

## 4. COVID-19 Vaccination Strategy Based on Cost-Effectiveness

Since the high-risk vaccination strategy is associated with lower costs and lower global health effects than the universal vaccination strategy, the optimal decision must be based on the comparison of their costs, health effects and cost-effectiveness. This analysis requires an assessment of effectiveness in preventing severe disease and SARS-CoV-2 infections, vaccination costs and disease costs associated with the universal and high-risk vaccination strategies.

Based on cost-effectiveness, the optimal decision is to implement the universal vaccination strategy when its incremental cost-effectiveness ratio is lower than the cost per Quality Adjusted Life Year (QALY) or Live Year Gained (LYG) that the public health system or the society are willing to pay [17]. The threshold cost-effectiveness ratio for this decision can range between EUR 30,000 and EUR 50,000 [17]. In the United Kingdom, the National Institute for Health and Care Excellence (NICE) used a threshold in the range of GBP 20,000–30,000 (USD 26,000–39,000; EUR 24,000–35,000) per QALY during 2000–2008 [18]. In Australia, the Pharmaceutical Benefits Advisory Committee (PBAC) has considered a threshold range of AUD 45,000 to AUD 60,000 per QALY [19]. The incremental cost-effectiveness ratio associated with the universal vaccination strategy is obtained by dividing the incremental cost by the incremental effectiveness. The incremental cost is the cost difference and the incremental effectiveness is the difference in effectiveness between the two strategies. The incremental cost-effectiveness of the universal vaccination strategy shows the cost of achieving an incremental unit of effectiveness (health effect) when the universal vaccination strategy is implemented instead of the high-risk vaccination strategy. When the incremental cost-effectiveness of the universal vaccination strategy is lower than the cost-effectiveness limit, the universal strategy must be implemented because it can increase the health effects achieved with the COVID-19 vaccination at a lower cost than the cost per effectiveness unit that the society is willing to pay.

In each country, the optimal COVID-19 vaccination strategy should be decided by assessing and comparing the cost-effectiveness of the universal and the high-risk vaccination strategies. A study carried out in the United States in 2023 assessing the cost-effectiveness of a universal vaccination program using updated adapted booster mRNA vaccines compared with primary vaccination only or booster vaccination without updated adapted vaccines found an incremental cost-effectiveness ratio of USD 33,437 per QALY in individuals aged 18 years or more, which was below the cost-effectiveness limit of USD 26,000–39,000 per QALY [20]. The incremental effectiveness ratios in different age groups were USD 115,588 per QALY in individuals aged 18–49 years, USD 25,000 per QALY in individuals aged 50–64 years and <USD 0 (cost saving) in individuals aged ≥65 years [20]. The study did not assess the cost-effectiveness in children. The study found that booster vaccinations for adults was a cost-effective intervention in the United States, although it was not cost-effective in individuals aged 18–49 years. Booster vaccinations for all adults or adults aged 50 years or more could, therefore, be a cost-effective intervention in other countries. However, the study did not assess the incremental cost-effectiveness of vaccinating all adults and vaccinating only high-risk adults.

## 5. Which COVID-19 Vaccines Should Be Used?

Deciding which COVID-19 vaccines should be used depends on the following factors: (1) emerging variants can escape the immune response generated by vaccines developed on the basis of the wild-type sequence of the SARS-CoV-2 spike protein (ancestral/original variant) [6,21,22], (2) the immune escape of an emerging variant is associated with lower vaccine effectiveness and greater viral transmissibility [8], and (3) new emerging variants can outdate adapted vaccines against prior variants.

COVID-19 vaccines generate high levels of antibodies and cellular immunity against the spike antigens (viral proteins) included in the vaccine, but emergent SARS-CoV-2 variants can escape the vaccine-induced neutralizing antibodies because their spike antigens have changed [6,22]. A study assessing the vaccine-induced neutralization activity of both primary and booster vaccinations using vaccines targeting the ancestral variant against Alpha, Beta, Delta and Omicron variants found lower neutralization activity against the Omicron variant than against the ancestral and prior variants, which was consistent with the immune escape associated with the Omicron variant [23].

Bivalent COVID-19 mRNA vaccines targeting the wild-type and Omicron BA.4/BA.5 (Pfizer-BioNTech vaccine, Cambridge, MA, USA) and BA.1 (Moderna vaccine, Cambridge, MA, USA) variants were used during 2022 and 2023, after the BA.5, BA.2 and XBB variants had replaced prior variants, to address the reduced effectiveness of COVID-19 monovalent vaccines during the Omicron variant predominance [21,24]. A meta-analysis of studies assessing the effectiveness of adapted vaccines targeting Omicron BA.1 and BA.4-5 variants found a vaccine effectiveness in preventing severe disease and SARS-CoV-2 infection of 56–60% and 36–39%, respectively [8]. COVID-19 vaccination programs should therefore use updated adapted vaccines.

The XBB variant originated from a recombination of two BA.2-derived variants, which replaced the BA.2 and BA.5 variants in 2023, and the JN.1 variant, which replaced the XBB variant in 2024 [25]. The WHO Technical Advisory Group on COVID-19 Vaccine Composition recommended the use of adapted vaccines targeting the XBB.1.5 variant in 2023 and targeting the JN.1 variant in 2024 [26]. However, vaccination programs should continue to use any of the WHO emergency-use-approved or prequalified COVID-19 vaccines, and vaccination should not be delayed in anticipation of access to vaccines with an updated composition [26].

By 17 September 2024, ten vaccines had been approved for emergency use by the WHO, European Medicines Agency (EMA) or Food and Drug Administration (FDA, Silver Spring, MA, USA) (Table 3). Only the Comirnaty (Pfizer/BioNTech), Spikevax (Moderna) and Nuvaxovid (Novavax) vaccines had been approved by the WHO, EMA and FDA (Table 3).

The WHO list of vaccines with emergency use authorization includes nine vaccines: Comirnaty (Pfizer/BioNTech), Spikevax (Moderna), Jcovden (Janssen, Beerse, Belgium), Nuvaxovid (Novavax, Gaithersburg, MD, USA), Covito (Beijing Institute of Biological Products, Beijing, China), Coronavac (Sinovac, Beijing, China), Covovax (Serum Institut of India, Pune, India), Convidecia (CanSino, Tianjin, China) and Corbevax (BioE, Seoul, Republic of Korea) [27] (Table 3). The EMA has authorized four vaccines for use in the European Union: Comirnaty (Pfizer/BioNTech), Spikevax (Moderna), Nuvaxovid (Novavax) and Bimervax (Hippra) [28] (3). The FDA has authorized three vaccines for use in the United States: Comirnaty (Pfizer/BioNTech), Spikevax (Moderna) and Nuvaxovid (Novavax) [29] (Table 3). Only the Comirnaty (Pfizer/BioNTech) vaccine targets the Omicron JN.1 variant [28].

Two of the vaccines approved by the WHO, EMA or FDA were developed using mRNA, three using non-replicating viral vectors, five using protein subunit particles and one using an inactivated virus (Table 3). RNA vaccines contain mRNA, produced in vitro using viral sequences, which directs cells to produce copies of harmless viral proteins. Non-replicating viral vector vaccines use a safe virus to deliver genetic material by coding a harmless protein (subunit) of the virus. Protein subunit vaccines contain harmless proteins (subunits) of the virus produced by engineered cell lines. Inactivated virus vaccines contain a harmless inactivated virus.

Approved COVID-19 vaccines are effective in preventing severe disease, but their effectiveness in preventing SARS-CoV-2 infection should be greater to prevent infection and block SARS-CoV-2 transmissibility [8,16]. Approved vaccines are associated with a lower effectiveness in preventing infection than that in preventing severe disease because they cannot generate a robust immunity at the respiratory mucosa, despite generating a robust humoral and cellular immune response [30]. Currently, there are 382 vaccine candidates, 183 vaccines in different clinical stages of development and 199 vaccines undergoing pre-clinical development, including DNA vaccines, RNA vaccines, inactivated virus vaccines, protein subunit vaccines, virus-like particle vaccines, live-attenuated vaccines, bacterial vector vaccines and viral vector vaccines [31]. The mucosal vaccines under development are based on different platforms, including live-attenuated vaccines, protein subunit vaccines, live-attenuated viral vector vaccines and inactivated viral vector vaccines [30]. Novel mucosal vaccines could reduce the infectivity of human secretions and reduce viral transmission by means of generating a localized and robust IgA-mediated immunity at mucosa surfaces [30,32].

## 6. Conclusions

In each country, the optimal COVID-19 vaccination strategy should be decided by considering the advantages and disadvantages and assessing the costs, health effects and cost-effectiveness of the universal and high-risk vaccination strategies. The optimal strategy can evolve depending on epidemic and economic considerations. The universal vaccination strategy should be implemented when the objective of the vaccination program is to achieve the greatest health benefits from COVID-19 vaccination and when its incremental cost-effectiveness ratio is lower than EUR 30,000–50,000 per QALY or LYG. The use of adapted vaccines targeting the currently circulating variants of SARS-CoV-2 is necessary to avoid the immune escape of emerging variants.

## Figures and Tables

**Table 1:** Booster COVID-19 vaccination strategies implemented in different countries for children, adolescents and adults in countries of the European Union and European Economic Area in the 2022−2023 vaccination campaign [12].

COVID-19 Vaccination Strategy	Countries of the European Union and European Economic Area
Children aged 5–11 years	
Universal strategy	Austria, Bulgaria, Malta, Poland
High-risk strategy	Belgium, Czechia, Estonia, France, Germany, Greece, Iceland, Ireland, Latvia, Netherlands, Norway, Portugal, Spain
Adolescents aged 12–17 years	
Universal strategy	Austria, Bulgaria, Cyprus, France, Germany, Hungary, Iceland, Ireland, Italy, Latvia, Liechtenstein, Lithuania, Luxembourg, Malta, Netherlands, Poland, Slovakia
High-risk strategy	Belgium, Czechia, Estonia, Finland, Greece, Norway, Portugal, Slovenia, Spain
Adults aged 18 years and above	
Universal strategy	Austria, Belgium, Bulgaria, Cyprus, France, Lithuania, Malta, Poland, Greece (30 years and above)
High-risk strategy (includes age ≥50–65 years)	Croatia, Czechia, Denmark, Estonia, Germany, Hungary, Iceland, Ireland, Finland, Latvia, Luxembourg, Netherlands, Iceland, Ireland, Liechtenstein, Norway, Romania, Portugal, Slovenia, Spain, Sweden

**Table 2:** Advantages and disadvantages of the universal and high-risk COVID-19 vaccination strategies.

	High-Risk Vaccination Strategy	Universal Vaccination Strategy
Advantages of the high-risk vaccination strategy		
Includes only individuals with the highest COVID-19 prevention necessity	Yes	No
Includes individuals without risk factors but moderate and low risk	No	Yes
Avoids vaccination adverse effects in individuals without risk factors	Yes	No
Inclusion among other medical interventions	Yes	No
Costs	++	+++
Advantages of the universal vaccination strategy		
Identification and communicationactivities are necessary	Yes	No
SARS-CoV-2 exposition reduction	No	Yes
Global health effects	++	+++
Herd immunity effects	+	+++

+++: very high effect, ++: high effect, +: moderate effect.

**Table 3:** COVID-19 vaccines approved for emergency use (5 September 2024) by the World Health Organization (WHO), European Medicines Agency (EMA) and Food and Drug Administration (FDA); SARS-CoV-2 variants included in vaccines, the vaccine platform and the target vaccination population.

Vaccine	Laboratory	Platform	SARS-CoV-2 Variant	Approved for Emergency Use			Population
				WHO	EMA	FDA	
Comirnaty	Pfizer-BioNTech	mRNA	Original	x	x	x	≥6 months
			Original BA.1	x	x	x	≥6 years
			Original BA.4-5	x	x	x	≥6 months
			XBB.1.5	x	x	x	≥6 months
			JN.1	x	x	x	≥6 months
Spikevax	Moderna	mRNA	Original	x	x	x	≥6 months
			Original BA.1	x	x	x	≥6 years
			Original BA.4-5	x	x	x	≥6 months
			XBB.1.5	x	x	x	≥6 months
Nuvaxovid	Novavax	Protein subunit adjuvanted	Original	x	x	x	≥12 years
			XBB.1.5	x	x	x	≥12 years
Bimervax	HIPRA	Protein subunit	Alpha Beta		x		>16 years
Jcovden	Jansen	Non-replicating viral vector	Original	x			
Covito	Beijing IBP	Inactivated	Original	x			
Coronavac	Sinovac	Inactivated	Original	x			
Covovax	Serum Institute of India	Protein subunit adjuvanted	Original	x			
Convidecia	CanSino	Non-replicating viral vector	Original	x			
Corbevax	BioE	Protein subunit	Original	x			

## Data Availability

No new data were created or analyzed in this study.
